# Groundwater potential assessment in the Blue Nile River catchment, Ethiopia, using geospatial and multi-criteria decision-making techniques

**DOI:** 10.1016/j.heliyon.2023.e17616

**Published:** 2023-06-24

**Authors:** Yohanis Tamesgen, Abunu Atlabachew, Muralitharan Jothimani

**Affiliations:** a Department of Geology, College of Natural and Computational Sciences, Arba Minch University, Arba Minch, Ethiopia; b Faculty of Water Resources and Irrigation Engineering, Water Technology Institute, Arba Minch University, Arba Minch, Ethiopia

**Keywords:** AHP, Blue Nile Basin, Ethiopia, Geospatial, Groundwater potential, Sensitivity analysis

## Abstract

Groundwater supplies have been exploited because of global water shortage. Therefore, effective management of water resources is crucial. Identifying potential groundwater regions in arid and mountainous terrains is challenging for many developing nations because of a lack of financial and human resources. An integrated strategy using remote sensing, geographic information systems, and multi-criteria decision analysis of the hierarchical analytical process was used to identify potential zones for groundwater in the Gulufa Watershed, Blue Nile River Basin, Ethiopia, which covers 1700 km^2^. Nine groundwater-influencing thematic layers were produced from conventional and satellite data, including lineament density, lithology, slope, geomorphology, soil, land use/land cover, drainage density, rainfall, and elevation. Satty scale values for the thematic layers and their classes were determined based on experts' opinions and literature. Thematic maps were integrated based on their weights and rates to produce a potential zone map using ArcGIS weighted overlay spatial function tool. According to the results, the prospect zone map consists of 383 km^2^ of very high, 865 km^2^ of high, 350 km^2^ of moderate, 58 km^2^ of low, and 0.3 km^2^ of poor zones. Validation of the potential zone map using existing boreholes yielded a close agreement, demonstrating the method's accuracy. According to the map removal sensitivity analysis results, the potential zone was more sensitive to lithology than other thematic layers. The map created in the research region can be an essential reference for identifying potential locations for additional groundwater resource exploration, planning, and management.

## 1 Introduction

The groundwater stored under the water table in the pores of soil and rock is one of the most critical and essential aspects of the natural hydrological cycle [1]. Its accessibility is vital for the domestic, agricultural, and socioeconomic development [2,3]. Population and urbanization pressures, the worldwide influence of climate change, recurring drought conditions, and a shortage of precipitation are driving an exponential increase in demand for groundwater [4]. Groundwater contributes to one-third of global freshwater withdrawal. It is a crucial and dependable water source in all climate zones, encompassing urban and rural areas in both developed and developing nations [4]. Most towns and communities in Ethiopia obtain household water from deep wells, boreholes, and shallow wells because of droughts, rural water supply, and irrigation. Groundwater has received increasing attention.

Most Ethiopian towns and villages acquire water for their domestic, agricultural, and industrial areas from surface runoff, represented by unprotected springs, rivers, ponds, and hand-dug shallow wells, which poses a significant health risk. The main reasons for this very low-performance level in the safe water supply are the lack of potential groundwater investigation and insufficient investment in the safe water supply. According to the International Atomic Energy Agency [5], more than 70% of Ethiopia's water supply comes from groundwater, although only 34% of the population has better drinking water. Thus, the nation requires immediate groundwater extraction and development.

However, the groundwater potential in Ethiopia is not well understood. Unviable groundwater use is a significant issue in many underdeveloped nations [6]. Groundwater is essential to Ethiopia's socioeconomic development, particularly in rural areas. The Gulufa watershed in the present study is located in West Wollega, a region widely known for farming and pastoral communities. A worse situation occurs when the rainy season is less than usual or during periods of complete drought when some people migrate to the densely populated highlands with their domestic animals. Most people in the study region travel great distances during the dry season in search of water and grazing pastures for livestock. However, no previous study has been conducted on the potential groundwater assessment in the present study region.

The following groundwater controlling parameters were considered in many studies to map the potential groundwater zones in different parts of the world: Lithological properties play a critical role in determining the porosity and circulation of the groundwater [12]. An increase in lithological porosity and permeability facilitates the storage and recovery of the groundwater. Various factors influence the hydraulic properties of an aquifer, including its permeability, hydraulic conductivity, and specific yield, as well as the physical properties of the rock, terrain, overlying unit, and weathering. Lineaments are structural elements relevant to groundwater [32]. Straight alignments of the structural, lithological, topographic, and drainage anomalies can exist. As a result of lineaments, surface runoff can be absorbed into the subsoil and thus be allowed to infiltrate and flow into the groundwater [12].

Runoff was positively correlated with slope steepness, whereas infiltration was negatively correlated with slope steepness. As the slope became steeper or more pronounced, the groundwater potential became less adequate [36,37,53]. The study area's groundwater infiltration capacity and soil moisture characteristics were determined using soil classification and texture parameters. The soil texture significantly influences the permeability of groundwater in a given area [36]. Land use/land cover (LU/LC) is a critical factor in watershed systems and significantly impacts infiltration, erosion, and evapotranspiration. LU/LC data are essential for identifying potential groundwater recharge areas [35,54]. The primary source of recharge is precipitation, followed by groundwater potential and all hydrological processes. In addition, the probability of water entering the aquifer was estimated [36]. When rainfall is abundant, the probability of groundwater is high; otherwise, the likelihood is low. Owing to regional and seasonal variations in precipitation, extensive research is required to determine the impact of rainfall on a given region. The drainage density, which has a strong inverse relationship with permeability, substantially affects the distribution of discharge and infiltration. A catchment with a greater drainage density produces a more significant discharge. However, a reduction in the drainage density of the catchment results in more substantial infiltration [15].

Zones with potential groundwater resources were identified using several conventional techniques, including geological, hydrogeological, geophysical, and photogeological methods. Since developing robust high-speed computers, digital techniques have combined numerous traditional methodologies with satellite imagery, remote sensing (RS) techniques, and geographic information systems (GIS). Recent studies have evaluated groundwater resources using GIS and RS tools [[7], [8], [9], [10]]. Remote sensing data provide a comprehensive view of observations' spatial and temporal distribution and save money and time [[11], [12], [13],^55^].

Studies using RS and GIS approaches to analyze groundwater potential differ significantly in terms of the type, number, and selection of thematic layers. Furthermore, most studies have evaluated various themes and their characteristics using subjective evaluations [14]. Numerous studies have shown that multi-criteria decision-making (MCDM) can improve the management of water resources by providing structure, auditability, transparency, and judgment rigor [15,17,18]. AHP is a technique for analyzing and ranking multiple complex decisions and determining the relative importance of factors. This method is widely recognized and appropriate for complex decision-making [50]. Based on the data provided, the effectiveness of these models was evaluated. Although other conventional procedures are effective and reliable, they are costly and time-consuming. AHP is, on the other hand, more efficient, cost-effective, and economical [51,52].

The AHP creates a set of numbers representing the relative relevance of each criterion through pairwise comparisons. Pairwise comparisons are well-established methods for calculating the relative weights of criteria. To evaluate all possible criterion pairs, weights represent a collection of weights defined by the eigenvector of a square reciprocal matrix [16]. The AHP model is a popular model used in groundwater potential assessment in different parts of the world [[17], [18], [19]]. Several studies have investigated the groundwater potential by integrating geospatial data and MCDM. Researchers in Ethiopia have studied groundwater potential and analyzed prospective groundwater zones using geographic information systems (GIS) and remote sensing techniques. Studies have been conducted on the Guna Tana landscape in Ethiopia's upper Blue Nile Basin [20], the Golina River Basin in the country's north [18], and the town of Arba Minch in the country's rift valley [21]. The western escarpment of the Ethiopian rift valley by Ref. [22], Ataye-watershed, Middle Awash Basin, Ethiopia [23], and Gerado River in Northern Ethiopia [6] examined the groundwater potential in different parts of the country.

The present study used GIS and MCDM approaches to determine the prospective groundwater zones of the Gulufa River watershed in Ethiopia's Blue Nile River basin. The objectives of this study are as follows:

1. To identify and delineate potential groundwater zones in the Gulufa River catchment,
2. To evaluate the degree and role of thematic layers in groundwater occurrence and distribution in the catchment; and
3. To validate the identified and delineated groundwater potential zones in the Gulufa River catchment.

The novelty of the present study are as follows:

➢ Integrated Approach: The research article contributes to the field by combining remote sensing, GIS, and multi-criteria decision analysis. This approach comprehensively assesses nine groundwater-influencing thematic layers, including lineament density, lithology, slope, geomorphology, soil, land use/land cover, drainage density, rainfall, and elevation. This provides a holistic understanding of potential groundwater zones.
➢ Application in Challenging Terrains: The study specifically focuses on the Gulufa Watershed, located in the arid and mountainous terrains of the Blue Nile River Basin in Ethiopia. By addressing the challenges associated with identifying potential groundwater zones in such regions, the study offers valuable insights for other developing nations facing similar constraints.
➢ Expert Opinion and Literature-based Satty Scale: The present research incorporates experts' opinions and existing literature to assign Satty scale values to determine the relative importance of thematic layers and their classes. This approach enhances the reliability and accuracy of the assessment, ensuring that the weights assigned to each thematic layer align with expert knowledge and relevant literature.
➢ Validation and Sensitivity Analysis: The potential zone map derived from the integrated analysis is validated using existing boreholes, demonstrating the accuracy and practical applicability of the method.
➢ Practical Implications: The resulting potential zone map is a valuable reference for identifying locations suitable for further groundwater resource exploration, planning, and management. By providing a comprehensive and reliable assessment of potential groundwater zones, the present study aids decision-makers in effectively managing water resources, particularly in regions with limited financial and human resources.

## 2 Materials and methods

### 2.1 Location of the study area

**Fig. 1:**
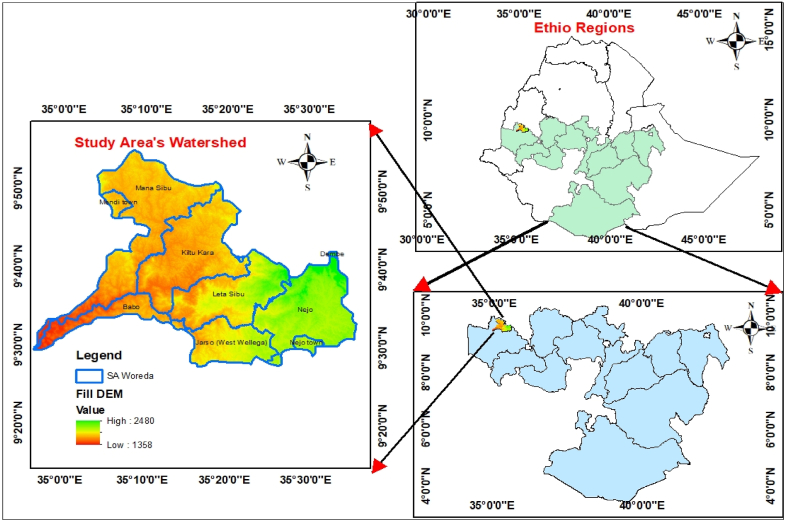
Location and elevation of the study area.

The Gulufa River catchment is situated in western Ethiopia within the Western Wellega Zone of the Oromia National Regional State. The administrative Woredas in the areas are Mana Sibu (Mendi Town), Jarso, Nejo, Kiltu kara, and Babo Gambel. The study area is approximately 386 km from Addis Ababa to Asossa. It is enclosed between 8° 00′00″N to 10° 00′00″N latitude and 34° 00′00″E to 36° 00′00″E longitude, covering an area extent of about 1700 km^2^ with an elevation range of 1358–2480 m a.s.l. (Fig. 1). This region is located at the intersection of the major local and national transportation routes that connect Addis Ababa, the country's capital, with other significant municipalities and towns, including Najo, Mendi, and Asossa. The overall topography of the Gulufa River catchment is characterized by its physical variance. The western and central regions, which constitute the catchment area, have flat or gently sloping topography.

### 2.2 Thematic layer preparation

Based on a literature review, nine groundwater regulation parameters were identified and used in the present study. These factors include elevation, rainfall, drainage density, slope, land use/landcover cover (LULC), lineament density, geomorphology, lithology, and soil type. These data were collected from various sources, and the groundwater inventory data were gathered for comparison. A Shuttle Radar Topography Mission digital elevation model (SRTM DEM) with a resolution of 30 m was used to create the drainage, slope, and elevation layers. A GIS environment was used to process the DEM and develop the thematic layers. A geomorphological map was created by manually digitizing different geomorphological characteristics and visually interpreting the relief map. The lithology and soil layers were produced by utilizing the areas already available from secondary data sources. The LULC and lineament of the study region were generated using a 10-m-resolution Sentinel-2 B image. Table 1 lists these data and their sources. The results and discussion provide a detailed explanation of the spatial distribution of individual thematic layers and their importance in determining groundwater potential.

**Table 1:** Summary of the source of the Data.

Data	Source of data	Data type/Year of sampling	Uses
SRTM DEM	https://earthexplorer.usgs.gov/	30 m * 30 m /2014	To generate slope, drainage, and Geomorphology.
Soil shape file	Ministry of Agriculture	Polygon /2016	Soil textural map
Geology map	Geological Survey of Ethiopia (GSE)	1:250,000 /2013	Geology map
Sentential 2B Satellite image	European Space Agency (ESA) (https://copernicus)	10 m * 10 m/2022	In order to create a lineament map and LULC map
Rainfall data	https://chrsdata.eng.uci.edu/	0.25° × 0.25° /	To prepare a mean annual rainfall map.
Existing water well data	MOWE and West Wellega zone water office	Wellpoint measurements /2010 to 2021 (average)	For validation of GWPZ maps

### 2.3 Methods

#### 2.3.1 The analytical hierarchy process (AHP)

The most prevalent GIS-based method for demarcating potential groundwater zones is the Analytical Hierarchical Process (AHP). A pairwise comparison matrix was created using AHP. The proportional weights of the respective classes were considered when calculating the cumulative weights of the primary criteria. Nine parameters were chosen for the potential groundwater assessment after the categorization and weighting of the maps. The weights were normalized to calculate the groundwater potential zone using the AHP approach, considering two themes and classes based on their relevance. Then, based on AHP [16], pairwise comparison matrices of given weights for various themes with subclasses were created. According to Ref. [16], the consistency ratio (CR) was calculated to assess the normalized weights of the different thematic layers and their classes.

All thematic layers were generated and integrated using the AHP technique based on their values and influences on groundwater potential. Several factors influence the presence of groundwater, including hydrological, geological, topographical, environmental, and climatic factors. It is tempting to try to determine which aspect or parameter impacts more or less in this circumstance. The following five procedures were used in this study to determine a suitable location for prospective groundwater using RS and GIS-based AHP analyses [14].

#### 2.3.2 Pairwise comparison of criteria

In the criteria comparison between pairs, the initial step was to compare the criteria pairwise, and the outcomes were then entered into a comparison matrix. A pairwise comparison matrix was created. As [16] suggested, each criterion was judged against the others based on how important it was on a scale from 1 to 9 (Table 2).

**Table 2:** Pairwise comparison scale.

Scale	AHP numeric value
Equally important	1
Equally to moderately important	2
Moderately important	3
Moderately to strongly important	4
Strongly important	5
Strongly to very strongly important	6
Very strongly important	7
Very strongly to extremely important	8
Extremely important	9

#### 2.3.3 Calculating criterion weights

Manually calculating the criterion weights entails performing the following steps [16]:

1. The pairwise comparison matrix's values in each column added up;
2. Dividing each matrix element by its column sum and called the normalized pairwise comparison matrix
3. The sum of the normalized scores for each row of the normalized matrix is multiplied by the overall number of criteria to determine the average of the elements in each row of the normalized matrix. AHP software was used in this investigation to compute the criterion weights.

#### 2.3.4 Calculation of principal eigenvalue vector

Adding the ratios of all the parameters and dividing by the number of parameters is known as the principal of eigenvalue (λ*max*). The principal of the eigenvalue must be equal to or greater than the number of parameters in the matrices; otherwise, the matrices must be rebuilt [16]. In this study, the principal eigenvalue of the 9 × 9 matrix was 9, which was used to compute the consistency index. AHP is a valuable tool for ensuring that decision makers' evaluations are consistent [16]. defined the consistency index as a measure of consistency and calculated it using the equation below (Eq. (1)).

(1) $$CI=\frac{\lambda \;\max-m}{m-1}$$

Where CI stands for consistency index, λ max is the eigenvalue and m for the number of thematic layers.

[16] suggested comparing the consistency index to the appropriate Random consistency index (RI). According to Ref. [16], the value of the Consistency Ratio must be less than or equal to 0.1 to accept consistency; otherwise, the judgment must be revised.

#### 2.3.5 Calculating consistency ratio (CR)

To find inconsistencies and create the best-fit weights in the entire pairwise comparison matrix, a consistency ratio (CR) was calculated after the pairwise comparison was completed, and the weight of the elements was established. The AHP analysis was continued with a consistency ratio of 0.1 or less [24]. However, if the consistency ratio is higher than 0.1, the evaluation process is inconsistent, and the AHP approach may not produce an accurate conclusion [24]. A consistency ratio was constructed for the matrix to assess the reliability of relative weights. The consistency ratio (CR) is a comparison of the consistency index (CI) and random consistency index (RI) and is calculated using the following (Eq. (2)):

(2) $$CR=\frac{CI}{RI}$$

where: CR, CI, and RI stand for consistency ratio, consistency index, and random consistency index, respectively.

#### 2.3.6 Groundwater potential index (GWPI)

GWPI is a dimensionless quantity that helps predict potential groundwater zones in a given area. After all thematic maps were developed, a GIS overlay analysis was conducted, and an AHP analysis was performed to determine the groundwater potential index (GWPI). The GWPI was calculated using a weighted linear combination approach [25], as shown in (Eq. (3)):

(3) $$GWPI=\sum_{j=1}^{m}\sum_{i=1}^{n}\left(Wj\;x\;Xi\right)$$

where **Wj** represents the normalized weight of the j thematic layer, **Xi** represents the rank/rate value of each class about the j layer, **m** represents the total number of thematic layers, and **n** represents the total number of classes in each thematic layer.

#### 2.3.7 Sensitivity analysis

Sensitivity analysis is used to quantify the degree of uncertainty or variability in the model output results [26,27]. Broadly, it assesses the resilience of a model when different input variables are used. Estimating the change in the output map with each change in the input enables us to comprehend the impact of each input parameter on the model's output. The effect of the input parameters on the model output depends on several factors, including the quantity, accuracy, and weights of the input parameters, the overlay type, and the rankings awarded. In the sensitivity analysis, the impact of each theme layer was quantified using index number S. The following equation (Eq. 4) was used to determine the sensitivity index.

(4) $$S=\frac{\left|\frac{GWPI}{N}-\frac{GWPI^{\prime }}{N^{\prime }}\right|}{GWPI}$$

where GWPI ′ and GWPI are the outputs of the Groundwater potential map index of when one of the thematic layers is removed and all the thematic layers, respectively. The number of all thematic levels utilized to calculate GWPI is N, and the number of thematic layers used to calculate GWPI ′ is N ′.

#### 2.3.8 Validation process

**Fig. 2:**
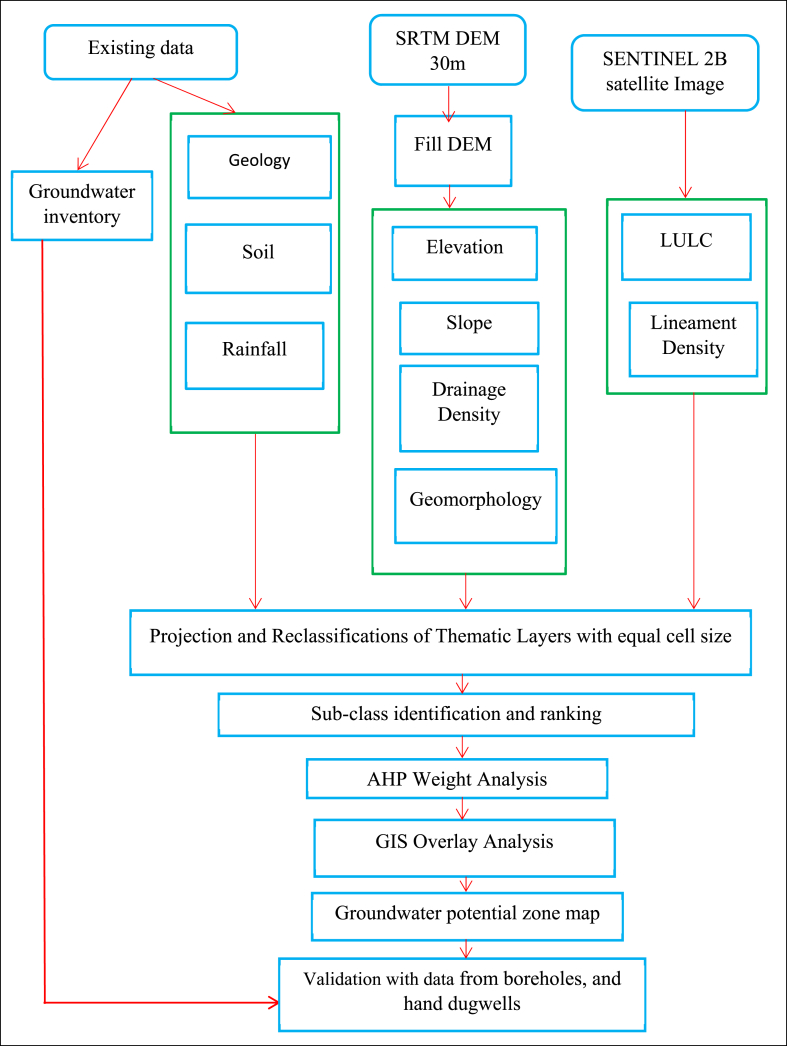
General methodological flow chart.

Data from hand-dug wells and boreholes in the study area were used to validate the resulting map of the potential groundwater zone. The data were prepared and placed on the study region's potential groundwater zone map. Groundwater potential zoning includes validation as a performance-evaluation method. A validation analysis was conducted using the well data for this study [28,29]. The ground data from hand-dug wells and boreholes were compared with the indicated potential groundwater zones using GIS and RS methodologies. The correlation was determined by comparing well data with a map. If there is poor correlation between them, the accuracy evaluation results in prompt additional rectification of the map of the indicated groundwater potential. The Ethiopian Ministry of Water and Energy and the West Wollega Zone Water Department gathered information on existing wells. The study area consists of 21 wells. The delineated map of GW potential was validated using data from drilled wells and ROC techniques. The overall methodology used in this study is illustrated in Fig. 2.

## 3 Results and discussions

### 3.1 Normalized weights for thematic layers

Nine thematic strata were assigned weights based on their significance for the groundwater potential. The analytical hierarchy process (AHP), eigenvectors, and normalized weights following this approach was used to calculate the normalized weights of the nine themes and their features. According to the AHP approach, the weighted values for the lithology were close to 30%, lineament density was 23%, the slope was 15%, geomorphology was 10%, the soil was 7%, LULC was 6%, rainfall was 4%, drainage density was 3%, and elevation was 2%. Lithology, as to weight vector calculations, is a well-known groundwater regulating parameter in catchment areas. However, elevation was given the lowest priority or ranking among the factors influencing groundwater potential in the study. Table 3 shows the pairwise comparison of the selected nine thematic layers, and Table 4 shows the final and weight % of the thematic layers.

**Table 3:** Pairwise comparison matrix for thematic layers.

Criteria	Lith	Ld	Slop	Geom	Soil	LULC	Rnf	Dd	Elvn
Lith	1								
Ld	1	1							
Slop	1/3	1/2	1						
Geom	1/4	1/3	1/2	1					
Soil	1/5	1/3	1/3	½	1				
LULC	1/6	1/4	1/3	½	½	1			
Rnf	1/7	1/5	1/4	1/3	½	1/2	1		
Dd	1/8	1/6	1/5	¼	1/3	1/2	1/2	1	
Elvn	1/9	1/7	1/6	1/5	¼	1/3	1/2	1/2	1

**Table 4:** Layer's weights.

Criteria	Weights	Weight %
Lithology	0.30254	30.254
Lineament density	0.22816	22.816
Slope	0.15038	15.038
Geomorphology	0.10082	10.082
Soil	0.06737	6.737
Land use/land cover	0.05856	5.856
Rainfall	0.03981	3.981
Drainage density	0.03005	3.005
Elevation	0.02221	2.221
Total	1	100

### 3.2 Lithology vs. groundwater potential

Lithology is one of the governing factors of groundwater that is included in groundwater investigations and substantially affects the extent and occurrence of groundwater [30]. Gimbi geological maps were used to create the lithological maps. The lithological features were clipped and converted to raster features for overlay analysis using a polygon-to-raster conversion tool. Lithology also influences the quantity and quality of underground water in a particular area [6]. According to Ref. [12], lithological characteristics affect the groundwater circulation and porosity. Higher lithological porosity and permeability resulted in better groundwater storage and increased groundwater yields. Aquifer permeability, hydraulic conductivity, specific yield, and other hydraulic properties are affected by the physical properties of the rock, the terrain nature, the overlying unit, the extent of weathering, and other factors.

**Fig. 3:**
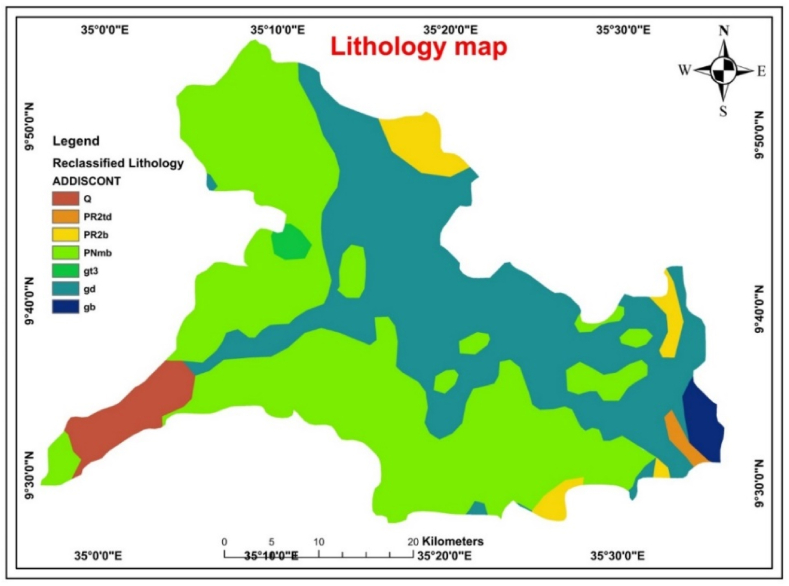
Lithology map **(**Where Q: Alluvial deposits, PR2td: Tulu Dimtu Group, PNmb: weathered basalts, PR2b: Birbir Group, gt3: weathered Late to post-tectonic granite, gd: Granodiorite, and gb: Gabbro**).**

Precambrian Metamorphic rocks, quaternary sediments, and tertiary rocks constitute most formations in this region (Fig. 3). These rock types were further classified into alluvial deposits, the Tulu Dimtu Group, weathered Makonnen basalt, the Birbir Group, weathered late-to post-tectonic granite, Granodiorite; and Gabbro. Grabens made by the western and eastern ridges expose alluvial sediments. Because of their excellent ability for infiltration and water recharge, the lithological class of quaternary alluvial deposit formations is where one can find ideal groundwater potential locations [31]. However, not all lithological units are crucial for identifying and managing groundwater. Basalt is the most extensively exposed volcanic rock type in the area. It constitutes the best aquifer next to alluvial deposits in the area. This high water-bearing capacity was structurally dependent.

Gabbroic complex rocks form a poor aquifer in which groundwater flow and storage are low within the low fracture zone, weathered zone, and through cooling or tectonic joints within the rock mass. The hydrogeological characteristics of these lithological units depend mainly on the secondary structures of the rock. The intensities of the secondary phenomena, such as jointing, shearing, faulting, and mineral alteration zones, were not uniformly distributed, as observed in the map. Therefore, these rock groups' water-bearing properties, permeability, and other hydraulic properties were highly variable. The granite unit is a mass of large granitic rocks with medium-to-coarse grain sizes. Groundwater was primarily collected from the fracture zone (cooling joints). Rock is typically regarded as a moderate aquifer. It generates moderate water storage and permeability zones, whereas low values were assigned for the Tullu Dimtu and Birbir groups for fracture, and faulting had less impact. The major component of the syn-to-late tectonic intrusion is the granodiorite unit. The large weathered section and some structural effects were mostly responsible for this unit's groundwater buildup and flow (NC36-12 Ghimbi and NC36-11 Tosho Terara sheets). A pairwise comparison of the lithology classes and AHP weights is presented in Table 5.

**Table 5:** Pairwise comparison matrix for lithology.

	AD	B	PD	TD	G	GD	BG	Weight
AD	1							31
B	1/2	1						22
PG	1/2	1/2	1					17
TD	1/3	1/2	1/2	1				12
G	1/4	1/3	1/2	1/2	1			8
GD	1/4	1/4	1/3	1/2	1/2	1		6
BG	1/5	1/4	1/4	1/3	1/2	1/2	1	4

Where AD = Alluvial deposit, B=Weathered Makonnen basalt, GD = Granodiorite, PG=Weathered late to post tectonic granite, TD = Tulu dimtu, G = Gabro, BG= Birbir Group.

### 3.3 Lineament density vs. groundwater potential

The lineament density of the watershed was defined as the sum of all the measured lineaments divided by the catchment area. Lineaments are the most important structural components relevant from the groundwater perspective [32]. It manifests as straight or curved linear alignment of structural, lithological, topographical, and drainage anomalies. These fissures aid the subsurface penetration of surface runoff and are crucial for groundwater flow and storage. Most geological linear features are located in areas where the bedrock is fractured and in states where it is porous and permeable, which can lead to the increased well output. PCI Geomatica software automatically derived a lineament density map of the Gulufa River catchments from Sentinel- 2B satellite images [33]. The main advantage of automatic lineament extraction over manual lineament extraction is that the same method can be used for many images, quick processing, and invisible lineaments. According to Ref. [34], lineament density is the length of lineaments in a specific area.

**Fig. 4:**
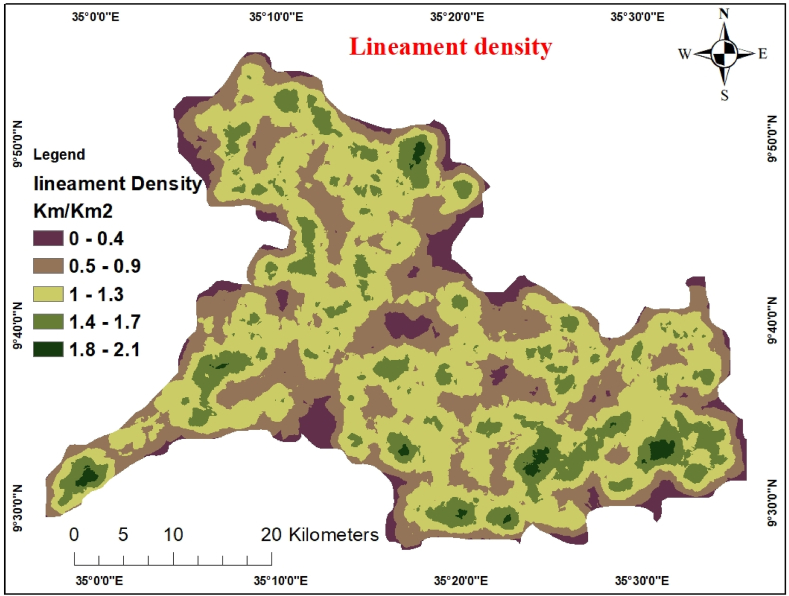
Lineament density map.

The line density function of the ArcGIS spatial analysis tool created a lineament density map of the Gulufa River catchment area. The equal-interval method was used to redistribute the lineament density map. Generating lineaments must be used to establish the direction of the groundwater circulation within the watershed. Those with a low lineament density are less favourable for groundwater recharge and discharge, whereas areas with a high lineament density are acceptable for both—jointed and sheared zones with good groundwater conduction. The lineament density map was reclassified into five categories, i.e., 1.8–2.1 (very high), 1.4–1.7 (high), 1–1.3 (moderate), 0.5–0.9 (low), and 0–0.4 (very low) density (Fig. 4). The pairwise comparison of lineament density classes and AHP weights are shown in Table 6.

**Table 6:** Pairwise comparison matrix for lineament density.

Lineament Density [Km/Km^2^]	1.8–2.1	1.4–1.7	1–1.3	0.5–0.9	0–0.4	Weight
1.8–2.1	1					51
1.4–1.7	1/3	1				26
1–1.3	1/5	1/3	1			13
0.5–0.9	1/7	1/5	1/3	1		6
0–0.4	1/9	1/7	1/5	1/3	1	3

### 3.4 Slope vs. groundwater potential

The slope is an essential topographical feature, explained by the contour area and horizontal spacing. Although every pixel in the elevation output raster carries an average value, sparse contours in vector form frequently exhibit milder slopes than closely spaced contours. The maximum rate at which the value changed from one cell to the next in the elevation raster was used to calculate the slope. The slope values are in degrees for both the vector and raster versions. The slope of the study area was constructed using ArcGIS software and the SRTM-30 m DEM resolution digital elevation model. The FAO classification system divides the slope map into five categories [35].

**Fig. 5:**
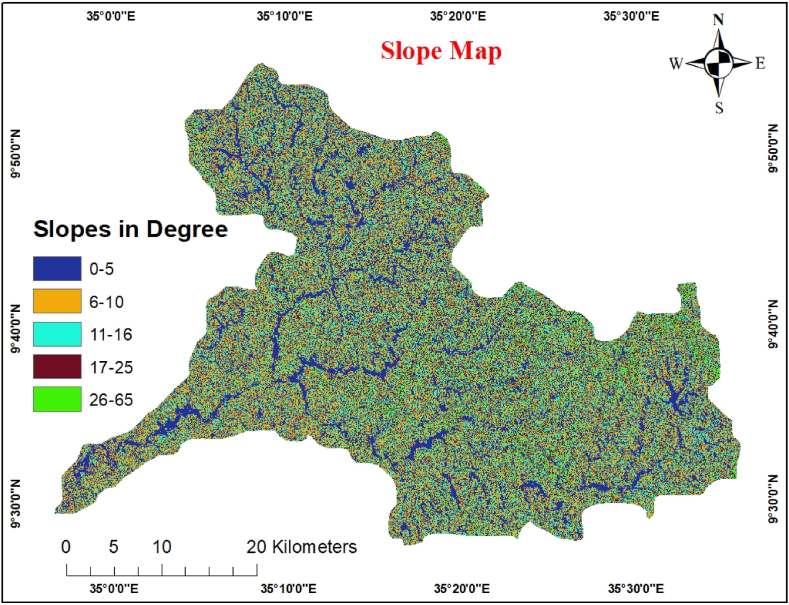
Slope map.

Lower slope values (gentle slope) imply smoother terrain, whereas higher slope values (sharp slope) suggest steeper topography. The slope is essential for determining the groundwater potential [36]. It controls the vertical percolation of water and surface runoff, affecting groundwater recharge [37]. The slope and infiltration exhibit opposite relationships [34,38]. The steeper the slope, the higher the runoff but, the lower the infiltration. As the slope becomes steeper or more extreme, the appropriateness of groundwater potential decreases. The scale value was provided in line with the fact that level terrain permits rainfall penetration and infiltration. At the same time, more significant slope areas are related to steeper slopes, which cause rapid runoff from land. The slope was then categorized into five classes using equal intervals:0–5 (high), 6–10 (moderate), 11–16 (moderate), and 26–65 (extremely low) (very high) [20]. Fig. 5 shows a slope map of the study area. The pairwise comparison of slope classes and AHP weights is shown in Table 7.

**Table 7:** Pairwise comparison Matrix for Slope.

Slope in degree	0–5	6–10	11–16	17–25	26–65	Weight
0–5	1					51
6–10	1/3	1				26
11–16	1/5	1/3	1			13
17–25	1/7	1/5	1/3	1		6
26–65	1/9	1/7	1/5	1/3	1	3

### 3.5 Geomorphology vs groundwater potential

Geomorphology is an essential factor in determining the potential of underground water. Once this characteristic has been assessed, it can be used to administer groundwater resources [37]. These are influenced mainly by geological formations [33]. The STRM DEM 30 m image and universal map downloader 3D satellite photos were used to update the main geomorphic features using the ArcGIS extension. Five landform classes were created [39]. The differences in elevation provide a hint for mapping the geomorphological features.

**Fig. 6:**
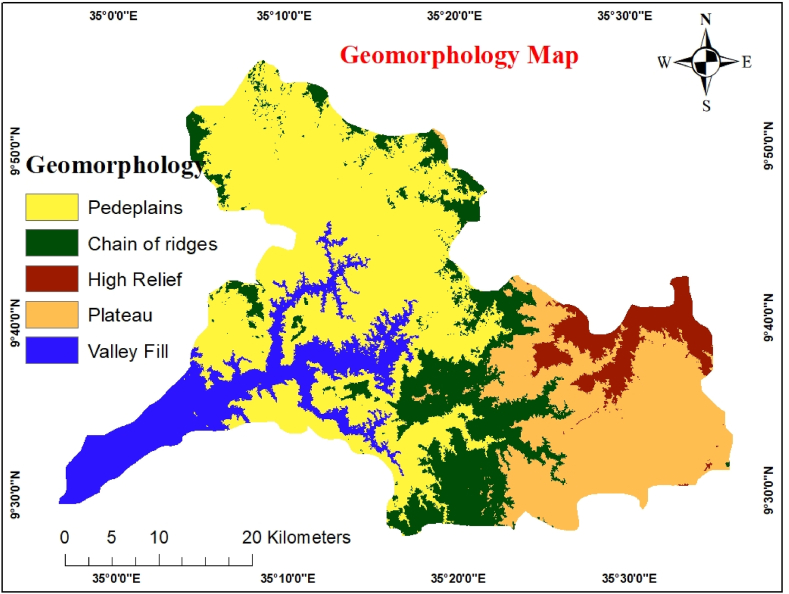
Geomorphology map.

The importance of geomorphology for groundwater resources and recharge processes depends on its properties and geographic heterogeneity. The region contains five geomorphological elements: valley fill, flat plains, low hills, plateaus, and mountains. Flat plain land appears in light yellow tones, and its uneven morphology is related to agricultural land producing good-to-moderate groundwater [39]. Mountains, residual hills, and landforms with unfractured rocks have a very low groundwater potential and infiltration. The impact of geomorphology, usually a dome-like and thick mass with sharp, deep narrow gorges, also negatively impacts groundwater storage, even if there is some rainwater percolation through the microfractures, columnar joints, etc. It facilitates rapid water flow, usually runoff, rather than storage. Pediplains and valley fill have high infiltration and good groundwater potential; hence, they are assigned higher ranks [39]. Moderate elevation variations and intermountain plains were observed here (Fig. 6). The pairwise comparison of geomorphology classes and AHP weights is shown in Table 8.

**Table 8:** Pairwise comparison Matrix for Geomorphology.

	VF	FP	LH	PL	HR	Weight
VF	1					51
FP	1/3	1				26
LH	1/5	1/3	1			13
PL	1/7	1/5	1/3	1		6
HR	1/9	1/7	1/5	1/3	1	3

Where VF= Valley fill, FP= Flat plain, LH = Low hills, PL= Plateau, HR= High relief.

### 3.6 Soil vs groundwater potential

The infiltration capacity of the region and soil moisture quality were determined using a soil classification map and soil texture factors. Groundwater permeability is significantly influenced by the soil texture prevalent in an area. Porous-structured soils are the best for promising groundwater potential because they easily permit surface water penetration and percolation into the subsurface, the interaction between soil quality and runoff and infiltration rates, and regulation of the permeability level. The FAO database of soil data for the study area includes information on the physical properties of the soil, such as its texture. Soil shape files of Ethiopian soil types were produced by the Ministry of Agriculture (MoA). The soil features were clipped and converted to raster using a polygon-to-raster tool for overlay analysis.

**Fig. 7:**
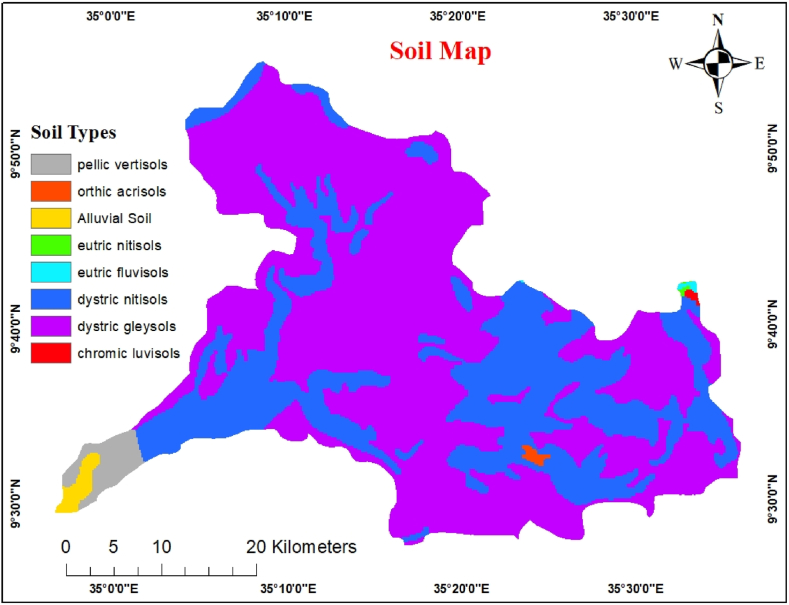
Soil map.

The soil in the study area varied from location to location concerning texture and thickness. The river basins in the study area are filled with thick alluvial and colluvial soils. Alluvial deposits are frequent and range in thickness around rivers, stream channels and flat plains. The catchment area has 7 different types of soil: Chromic luvisols, Dystric gleysols, Dystric nitosols, Eutric fluvisols, Eutric nitosols, Orthic acrosols, and Pellic vertisols. Eutric Nitosols, Chromic Luvisols, Orthic Acrosols, and Eutric Fluvisols cover the study regions (Fig. 7). The pellic vertisol is the most prevalent soil type in the region and primarily encompasses the lowlands. Dystric nitosols are the region's second most prevalent soil type, primarily encompassing the lowlands and highlands. Chromic luvisols were found in a few isolated locations in the highlands. All soil types were ranked according to their groundwater transmissivity based on various factors, as Gumma and Pavelic (2013) recommended. The correlation between runoff and absorption rates, which regulates permeability, the key hydrogeological factor determining groundwater potential, is influenced by soil texture. In the study area Dystric Nitosol, Chromic Luvisol, Eutric Fluvisol, and Orthic Acrosol soil types are classified into loamy soil texture, Dystric Gleysol is classified into sandy clay, and Eutric Nitosol, and Pellic Vertisol are classified into sandy loam, and clay soil textures, respectively.

The lowest value was obtained for clayey soils because the clay layers severely limit the percolation. In contrast, the most significant value was given to the sandy loam because of its high permeability and rapid percolation. The pellic vertisol was fine-grained clay soil, indicating poor groundwater potential. Water can enter coarse-grained soil more quickly than fine-grained soil because of the connectivity gap between the porous media (Hussein et al., 2016). As a result, Eutric Nitosol, Chromic Luvisol, Eutric Fluvisol, and Orthic Acrosol are medium-grained soils with moderate groundwater potential. At the same time, Dystric Nitosol is a coarse-grained soil with good groundwater potential. The pairwise comparison of soil classes and AHP weights are shown in Table 9.

**Table 9:** Pairwise comparison matrix for soil.

	EN	DG	DN	CL	EF	OA	PV	Weight
EN	1							38
DG	1/3	1						25
DN	1/5	1/3	1					16
CL	1/5	1/5	1/3	1				9
EF	1/5	1/5	1/5	1/3	1			6
OA	1/5	1/5	1/5	1/5	1/3	1		4
PV	1/7	1/5	1/5	1/5	1/5	1/3	1	2

EN= Eutric Nitosol, DG = Dystric Gleysol, DN= Dystric Nitosol, CL= Chromic Luvisol, EF= Eutric Fluvisol, OA= Orthic Acrosol, PV= Pellic Vertisol.

### 3.7 LULC vs. groundwater potential

Land use is a crucial component of a watershed system that influences infiltration, erosion, and evapotranspiration. To determine areas with potential for groundwater recharge, a land use/cover map is one of the key regulatory elements [6]. Land use significantly influences the development of groundwater resources. The surface cover makes the surface rougher, which increases infiltration while lowering discharge. In contrast to urban regions, where runoff is likely to increase, forested areas have higher infiltration and less runoff. Remote sensing delivers superior data on the distribution pattern of vegetation cover and land use in less time and at a lower cost than traditional methods. Using supervised classification from Sentinel- 2B satellite image and the maximum-likelihood algorithm, the LULC map was finally created.

**Fig. 8:**
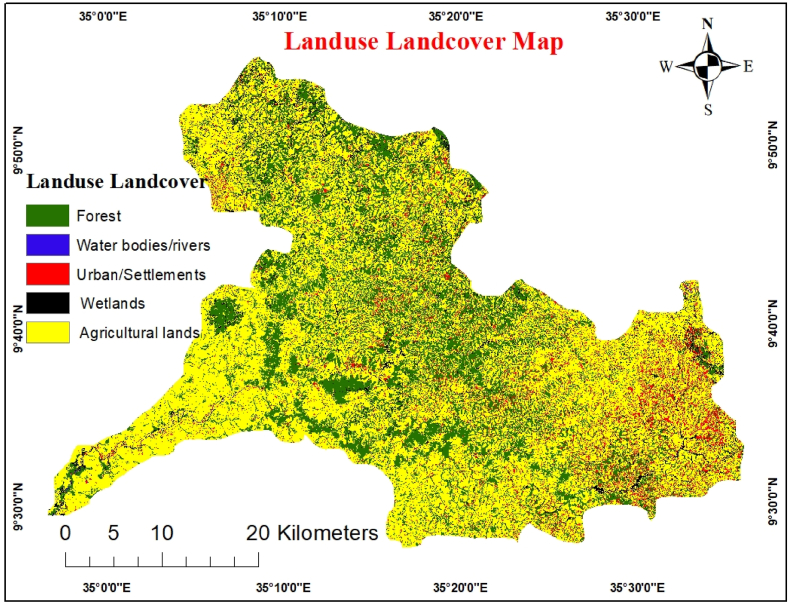
LULC map.

Five significant LULC units were identified in this study. Most research areas include cultivated/agricultural land, forests, wetlands, water bodies, and residential areas. The dominant 60% of the Gulufa watershed LULC is composed of agricultural units, followed by forests and settlements, which account for 28% and 6.8% of the total area, respectively (Fig. 8). Built-up and barren surfaces are less likely to contain groundwater than vegetation-covered regions, such as those covered in agricultural plants and forests [40,41].

Although some land uses negatively affect groundwater resources and recharge, others have positive effects. For instance, runoff is mainly produced in inhabited and urban regions, which reduces recharge. Depending on the management strategies used for soil and water conservation, agricultural regions may or may not positively affect the groundwater. The built-up area was the land-use class with the lowest weight relative to the other groups. However, forests reduce runoff, increasing the possibility of groundwater recharge. The primary sources of improved underground water are wetlands and bodies of water. However, forests reduce the runoff process, increasing the possibility of groundwater recharge. The primary sources of improved underground water are wetlands and bodies of water. Priorities for LULC units were as follows, in order of groundwater suitability: urban/settlement land > agricultural land > forest > water body [42]. The pairwise comparison of LULC classes and AHP weights is shown in Table 10.

**Table 10:** Pairwise comparison matrix for LULC.

	WB	WL	FR	AL	S	Weight
WB	1					51
WL	1/3	1				26
FR	1/5	1/3	1			13
AL	1/7	1/5	1/3	1		6
SE	1/9	1/7	1/5	1/3	1	3

Where, WB- Water bodies, WL- Wetlands, FR- Forest, AL- Agricultural lands, SE- Settlement.

### 3.8 Rainfall vs. groundwater potential

The primary recharge sources were rainfall, groundwater potential, and hydrological processes. In addition, it estimates the possibility of seepage into an aquifer system [43]. The hydrological cycle, essential to the hydrological cycle, is mainly influenced by rainfall. This is vital to the natural processes that control groundwater potential. Long-term rain is more likely to reveal substantial groundwater recharge than short-term rainfall, which suggests a low groundwater recharge [43]. The data was downloaded from 2010 to 2021 from the https://chrsdata.eng.uci.edu/.

**Fig. 9:**
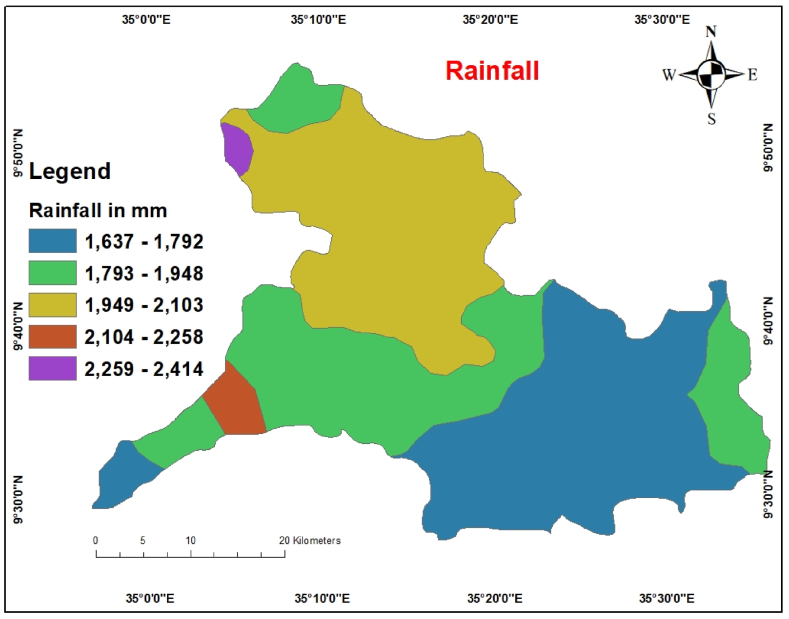
Rainfall map.

The average yearly rainfall data of the research area were produced using the IDW interpolation method and then classified into five categories (Fig. 9) based on the value of utilizing natural breaks. If the rainfall is excellent, the likelihood of groundwater is high, and if the rainfall is low, the probability is low. Because rainfall varies both regionally and seasonally, a detailed investigation is required to determine the effects of rainfall in any particular region. Thus, when examining groundwater suitability, places with high rainfall amounts were assigned a greater weighted value. The northwestern region of the study area has comparatively heavy rainfall, whereas the southeastern region has a relatively low amount. The research area experiences average rainfall in the range of 1637–2414 mm, which is relatively high for the entire watershed. The pairwise comparison of rainfall classes and AHP weights are shown in Table 11.

**Table 11:** Pairwise comparison Matrix for Rainfall.

	2259–2414	2104–2258	1949–2103	1793–1948	1637–1792	Weight
2259-2414	1					51
2104-2258	1/3	1				26
1949-2103	1/5	1/3	1			13
1793-1948	1/7	1/5	1/3	1		6
1637-1792	1/9	1/7	1/5	1/3	1	3

### 3.9 Drainage density vs groundwater potential

Drainage density measures how closely spaced the closeness of stream channels to the total length of all stream segments within a catchment [44]. The drainage density reveals the rock permeability and infiltration capacity and determines the recharge capacity. This is a regulatory factor in zones where groundwater may be present. Drainage density, which has a strong inverse relationship with permeability, significantly influenced the distribution of runoff and infiltration. The runoff will be higher if the catchment drainage density is higher. However, infiltration will be higher if the catchment drainage density is lower.

**Fig. 10:**
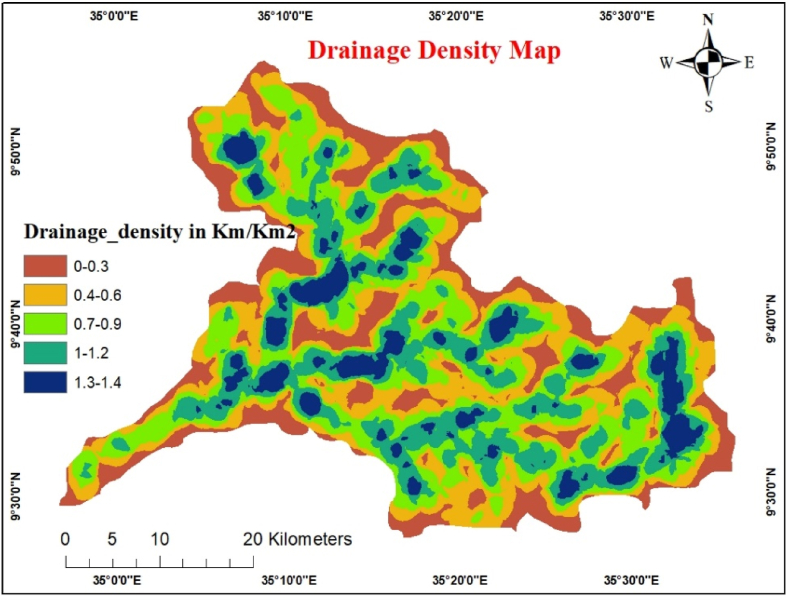
Drainage density map.

SRTM DEM 30-m data were used to generate the watershed drainage using the Strahler method in a series of steps. The ArcGIS spatial analyzer line density tool utilized the polyline stream as an input to create drainage density. Using equal intervals, drainage density was classified into five classes (Fig. 10). A potential groundwater zone could also occur. In addition, the tectonic process of the region, along with its geological variations, affects the drainage pattern and density. Most young lineaments serve as channels for rivers. The drainage density and level of gorges formed by rivers or streams vary from place to place based on rock variation and structural impact. The drainage density was then divided into five parts using an equal interval of 0–0.3 (very high), 0.4–0.6 (high), 0.7–0.9 (moderate), 1–1.2 (low), and 1.3–1.4 (very low) density (Kumar et al., 2017). Consequently, there may be a potential groundwater zone. A pairwise comparison of the drainage density classes and AHP weights is presented in Table 12.

**Table 12:** Pairwise comparison matrix for drainage density.

	0–0.3	0.4–0.6	0.7–0.9	1–1.2	1.3–1.4	Weight
0–0.3	1					51
0.4–0.6	1/3	1				26
0.7–0.9	1/5	1/3	1			13
1–1.2	1/7	1/5	1/3	1		6
1.3–1.4	1/9	1/7	1/5	1/3	1	3

### 3.10 Elevation vs. groundwater potential

**Fig. 11:**
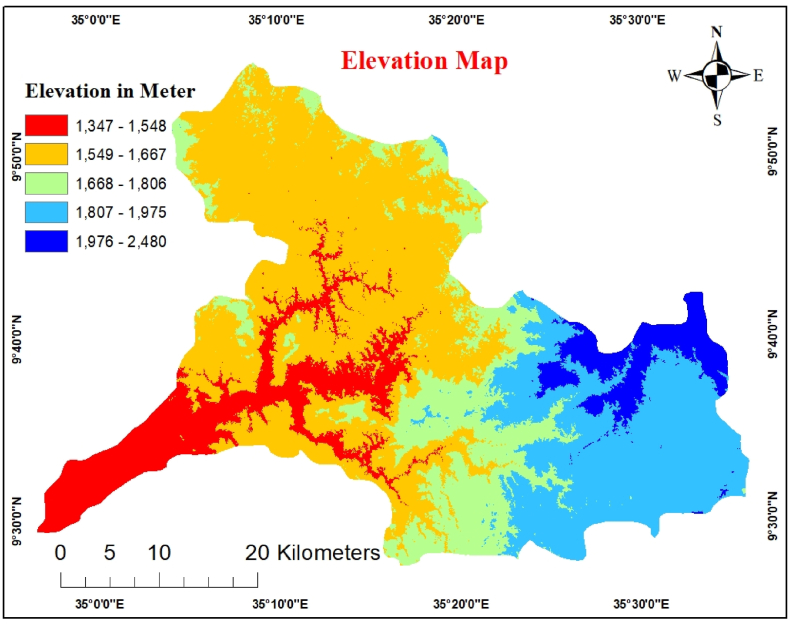
Elevation map.

The elevation was calculated using the SRTM DEM 30-m 30-m spatial resolution data because of its accurate vertical and lateral results [33]. The elevation of the Gulufa Watershed was plotted using ArcGIS. Additionally, slope raster data for each grid cell were created using elevation data. High elevations promote recharge and ensure groundwater availability in the lowland sections of watersheds. There is a deeper water table at the stream divide (ridge) than below the floodplain [44]. The suitability of groundwater decreased as the elevation increased. The lower topography stores more water than the higher terrain. An elevation map of the study area is shown in Fig. 11. A pairwise comparison of the elevation classes and AHP weights is presented in Table 13.

**Table 13:** Pairwise comparison matrix for elevation.

	1347–1548	1548.1–1667	1667.1–1806	1806.1–1975	1975.1–2480	Weight
1347–1548	1					51
1548.1–1667	1/3	1				26
1667.1–1806	1/5	1/3	1			13
1806.1–1975	1/7	1/5	1/3	1		6
1975.1–2480	1/9	1/7	1/5	1/3	1	3

### 3.11 Groundwater potential zoning

Groundwater potential zones were established using remote sensing and Geographic Information System (GIS) approaches. The nine thematic map overlay analyses were layered using ArcGIS Software to obtain the groundwater potential index. A dimensionless number called GwPI predicts the locations of groundwater potential zones. According to (Eq. (3)), the weighted linear combination method was used to estimate GwPI [43]. The total number of classes in a thematic layer, m is the total number of thematic layers, n is the total number of classes in the jth thematic layer, and Xi is the rate value of each class in the jth layer.

Weighted overlay analysis was used to map the potential zones of groundwater. The input datasets comprised nine raster-formatted thematic maps that were classified. Each factor's allocated weight is placed on a scale. The overlay analysis was carried out in Arc GIS 10.8.1 software by using the following (Eq. 5).

(5) GWPZM=0.3Lith+0.23Ld+0.15Sl+0.10Gm+0.07So+0.06LULC+0.04Rf+0.03Dd+0.02Elv

where Elv stands for elevation, Sl for slope, Ld for lineament density, Lith for lithology, and LULC for land use and land cover, S stands for “soil,” Dd “drainage density,” Rf “rainfall,” and Gm “geomorphology."

**Fig. 12:**
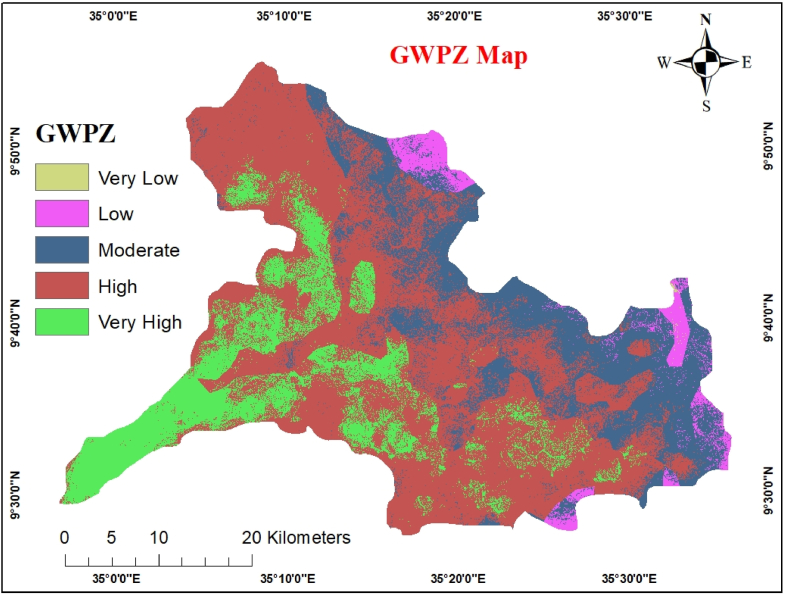
Groundwater potential zones.

Groundwater potential mapping does not have a standardized classification system that specifies low or high potential [45]. Utilizing the spatial analysis tool in ArcGIS 10.8.1, all nine thematic layers for the study were overlaid in a raster format. The high groundwater potential zone, mainly found in the middle to lower half of the watershed, is shown in the output map (Fig. 12). This is primarily due to the quaternary alluvial sediment aquifer in plain terrain. Only a few other areas with substantial groundwater potential have lineament densities that range from high to extremely high and have a mild slope.

The high groundwater potential zone covers 865 km^2^ of the Gulufa Watershed. Around the water-bearing geological unit are the moderate groundwater potential zone, moderately sloping terrain, almost level plains, plains with chains of hills, and soils with eutric regosols. A minor portion of the intermediate groundwater potential zone also spreads along a direction with a relatively high lineament density. The moderate groundwater potential zone covers 350.5 km^2^ of the Gulufa watershed, a sizeable area. Low groundwater potential areas are found in the northern and northeastern parts of the research area, which together make approximately 58 km^2^ of the watershed.

This results from the area's high concentration of hilly geomorphology and relatively low abundance of lithological formations that contain water. The characteristics linked to low-slope and high-lineament density regions can be observed in alluvial plains, quaternary sediments, fracture valleys, and valley fills. Most groundwater potential zones with low and poor quality are composed of structural hills and escarpments, mountain peaks, plateaus, and regions with low fractured rock formation [33]. The potential groundwater zones in the current study are shown in Fig. 12.

### 3.12 Sensitivity analysis

Sensitivity analysis is a method for quantifying the degree of variation in model output results [26]. A sensitivity analysis was performed on the map removal process. Estimating the change in the output map with each change in the inputs enables us to comprehend the impact of each input parameter on the model's output. The influence of the input parameters on the model output depends on various factors, including the quantity, accuracy, weights, and ranks of the input parameters and the type of overlay used [46].

Nine thematic layers were used to process the output map, and one thematic layer was removed. Simultaneously, one thematic layer was removed. The output map changed when a layer was removed, demonstrating that each thematic layer used in this AHP technique, despite having a different mean variation index value, serves a particular purpose in GWP delineation. Table 14 lists the values of the computed sensitivity indices. The output map of the potential groundwater zones of the Gulufa watershed was determined in this study by the lithological thematic layer (Sde = 23), the first and most significant one [48]. Additionally, the distribution of GWP zones was extremely sensitive to rainfall (Sde = 21), indicating that rain was a key factor affecting this distribution. However, slope (Sde = 19.8) had the most negligible impact on the decision to produce a map (i.e., least significant) [48].

**Table 14:** Sensitivity index.

Layer Removed	Variation Index			
	Minimum	Maximum	Mean	Std. Deviation
Elevation	0.063191	53	20	20.9
Lineament	0.009972	44	20	20.5
Geomorphology	0.018544	52	20	20.7
LULC	0.094528	51	20	20.4
Slope	0.046709	50	20	19.8
Rainfall	0.026403	54	19	21.5
Drainage	0.090621	52	20	20.6
Soil	0.361761	52	20	20.3
Lithology	0.037546	48	20	23.2

### 3.13 Validation analysis

**Fig. 13:**
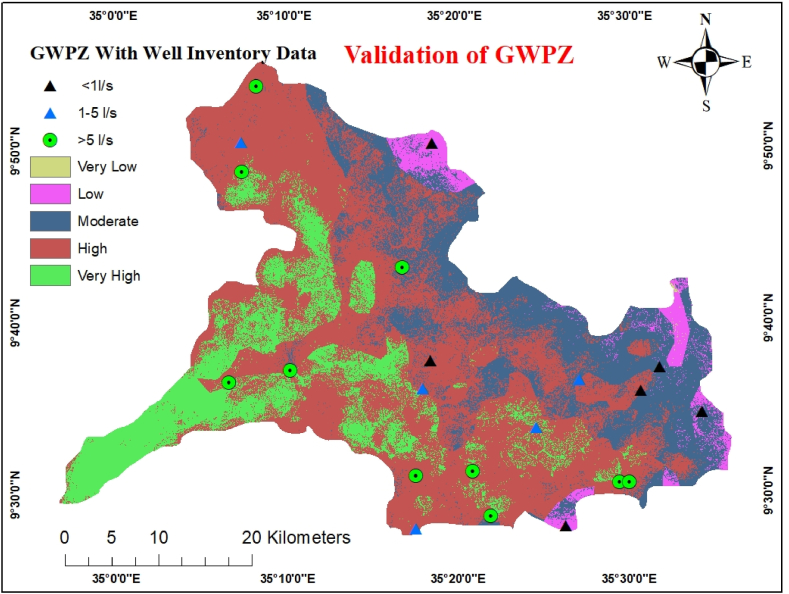
Validation of GWPZ with Well data.

**Fig. 14:**
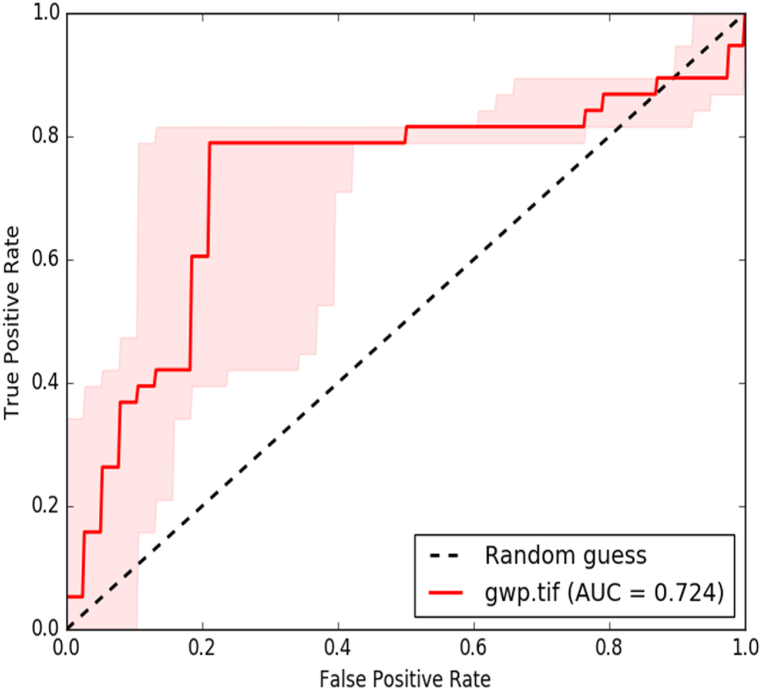
Area under curve.

The acquired well data verified the accuracy of the determined potential groundwater zone [9]. The analysis findings were validated by superimposing the drill yield data of the research area on a map of the categorized predicted potential groundwater zones. Qualitative validation was performed by directly comparing the yield data from various classes using a potential map (Fig. 13). The quantitative validation process involving the creation of receiver operating curves (ROC) is fairly reliable (Fig. 14). The ROC curve, which indicates the degree of accuracy of the constructed model for estimating the groundwater potential, was quantitatively assessed using the Area Under Curve (AUC).

The model was considered adequate if the AUC exceeded 0.7 [48]. The relationship between AUC and prediction accuracy can be summarized as follows [47]: Poor (0.5–0.6), average (0.6–0.7), good (0.7–0.8), very good (0.8–0.9), and excellent (0.9–1). Approximately 41 existing well data points were collected, including hand-dug wells and deep and shallow wells drilled by different organizations. Out of the 41 bore wells drilled in the general neighbourhood, 21 matched the predicted map, and 20 did not. With the point-source inventory data, identifying the groundwater potential zone using integrated GIS and remote sensing approaches has an AUC of approximately 72.4% (Mukherjee et al., 2020). The quantitative model (ROC) was considered acceptable because the AUC was greater than 0.7 (Fig. 14). As a result, the outcome of the analysis reveals that most of the evaluations were confirmed to match the data gathered.

The groundwater inventory wells were classified as 1, 1–5, and >5 l/s during the validation analysis to satisfy the corresponding groundwater potential zone classifications [49]. This demonstrates that the groundwater point data and potential groundwater zones identified by the GIS and RS methods are in close agreement. The light green circle in Fig. 13, which depicts the high class on the map, indicates the point where the water sources have high-class potential. Highly concentrated zones with high groundwater potential can be found in the region, particularly in quaternary alluvial sediments near the river. In Fig. 13, the black triangles represent the potential low-class status, whereas the blue triangles represent the potential moderate-class status. The very low class did not contain any data compiled from point sources. The Quaternary sediment aquifer and high lineament density were present in the zones with the lowest slope values, as seen by the abundance of very high-yield water on the verification map sites (>5 l/s) (Fig. 13). On the other hand, areas with very low and low groundwater potential (<1 l/s) are distinguished by the presence of mountains, complicated landforms, and slopes that range from moderately to extremely steep.

## 4 Conclusions

The Gulufa watershed in the present study is located in West Wollega, a region widely known for farming and pastoral communities. A worse situation occurs when the rainy season is less than usual or during periods of complete drought when some people migrate to the densely populated highlands with their domestic animals. This study used RS, GIS, and AHP methodologies to identify potential groundwater zones. Using these nine thematic layers as proxies for groundwater, this study attempted to create a spatial model for defining potential zones. These include geology/lithology, lineament density, slope, geomorphology, soil, LULC, rainfall, drainage density, and elevation. The high groundwater potential zone covers 865 km^2^ of the Gulufa Watershed. Around the water-bearing geological unit is the moderate groundwater potential zone, moderately sloping terrain. A minor portion of the intermediate groundwater potential zone also spreads along a direction with a relatively high lineament density. The moderate groundwater potential zone covers 350.5 km^2^ of the Gulufa watershed, a sizeable area. Low groundwater potential areas are found in the northern and northeastern parts of the research area, which together make approximately 58 km^2^ of the watershed. The five different groundwater potential zones—very high, high, moderate, low, and very low (poor)—were validated using underground water inventory data already used in the study region. The results of the validation statistics are satisfactory. AHP's AHP (AUC = 0.724) accuracy was good based on the ROC validation results. The AHP method is one of the most appropriate techniques for assigning weights to groundwater prospect studies because of the accuracy of the results. The sensitivity analysis shows the lithological thematic layer (Sde = 23), the first and most significant one in the occurrence of groundwater in the present study area.

Additionally, the distribution of GWP zones was extremely sensitive to rainfall (Sde = 21), indicating that rain was a key factor affecting this distribution. However, slope (Sde = 19.8) had the most negligible impact on the decision to produce a map (i.e., least significant). Overall, this study shows the importance of remote sensing, GIS, and AHP methods in groundwater prospecting in the present study area, and the outcomes of this study can be used in future research. The groundwater potential zone map of the present study provides information to water stakeholders, local governments, and decision-makers to develop enhanced sustainable water use for urban and agricultural purposes in this region, thereby reducing costs, time, and human effort. This study's primary limitations were the lack of complete groundwater inventory data and the irregular distribution of data points.

## Author contribution statement

Yohanis Tamesgen: Conceived and designed the experiments; Performed the experiments; Analyzed and interpreted the data; Contributed reagents, materials, analysis tools and data; Wrote the paper.

Abunu Atlabachew: Conceived and designed the experiments; Performed the experiments; Analyzed and interpreted the data; Wrote the paper.

Muralitharan Jothimani: Analyzed and interpreted the data; Contributed reagents, materials, analysis tools and data; Wrote the paper.

## Data availability statement

Data included in article/supp. material/referenced in article.

## Declaration of competing interest

The authors declare that they have no known competing financial interests or personal relationships that could have appeared to influence the work reported in this paper.
